# RE-AIM EVALUATION OF A VIRTUAL “AGING WITH PAIN” INTEGRATIVE HEALTH EDUCATION PROGRAM DURING THE COVID-19 PANDEMIC

**DOI:** 10.1093/geroni/igac059.3072

**Published:** 2022-12-20

**Authors:** Jennifer Brailsford, Sophia Sheikh, Jason Beneciuk, Monika Patel, Brittany Johnson, Robin Moorman Li, Phyllis Hendry, Natalie Spindle

**Affiliations:** University of Florida, Jacksonville, Florida, United States; University of Florida College of Medicine – Jacksonville, Jacksonville, Florida, United States; University of Florida, Gainesville, Florida, United States; University of Florida College of Medicine – Jacksonville, Jacksonville, Florida, United States; UF Health Jacksonville, Jacksonville, Florida, United States; University of Florida College of Pharmacy, Jacksonville, Florida, United States; University of Florida College of Medicine – Jacksonville, Jacksonville, Florida, United States; University of Florida College of Medicine – Jacksonville, Jacksonville, Florida, United States

## Abstract

Literature suggests integrative pain management strategies reduce chronic pain and opioid use. However, many older adults are unaware of these options. The Aging and Integrative Pain Assessment and Management Initiative (AI-PAMI) launched in 2020, providing webinars and recorded presentations on integrative pain management for adults > age 50, caregivers and healthcare providers. The RE-AIM (Reach, Effectiveness, Adoption, Implementation, Maintenance) framework was used to evaluate AI-PAMI via the following measures: participant demographics, survey results, program elements and qualitative findings. Reach: There have been > 20,000 views of recorded content and 48% (885/1,859) of registrants attended a live webinar. Effectiveness: Survey results demonstrate 75% of providers and 73% of older adults/caregivers reported new knowledge gain; and 80% of providers and 60% of older adults/caregivers reported changing their pain management practice/routine. Adoption: Presentations were delivered by 33 multidisciplinary experts from 12 different institutions. Six regional stakeholders promoted AI-PAMI using their dissemination networks. Implementation: The COVID-19 pandemic changed program delivery from an in-person model to virtual. To date, AI-PAMI has delivered 17 live webinars and 25 recorded presentations. Live webinars are delivered with a didactic, Q&A discussion and follow-up email. To refine AI-PAMI, 11 healthcare providers and 16 older adults participated in focus groups or in-depth interviews. Maintenance: AI-PAMI is in its third year and will be maintained under a long-standing institution-wide program. Website content will be sustained and remain free access. AI-PAMI is a valuable educational resource for older adults, caregivers, and healthcare providers. Virtual delivery is accommodating for a post-COVID environment.

